# Temporal Evolution of Intrapartum Fetal Heart Rate Features

**DOI:** 10.23919/cinc53138.2021.9662865

**Published:** 2021-09

**Authors:** Johann Vargas-Calixto, Yvonne Wu, Michael Kuzniewicz, Marie-Coralie Cornet, Heather Forquer, Lawrence Gerstley, Emily Hamilton, Philip Warrick, Robert Kearney

**Affiliations:** 1 McGill University, Montreal, Canada; 2 University of California, San Francisco, USA; 3 Kaiser Permanente, Northern California, USA; 4 PeriGen Inc., Montreal, Canada

## Abstract

Our research goal is to improve the intrapartum identification of tracings associated with severe acidosis at birth and subsequent hypoxic-ischemic encephalopathy so that timely interventions could avoid such complications without causing excessive unnecessary interventions in births with normal outcomes. The present study examines the evolution of fetal heart rate (FHR) features over the course of labor. We analyzed FHR signals collected in the last 6 hours before delivery in 21,853 births with normal neonatal outcomes and in 163 that developed hypoxic-ischemic encephalopathy (HIE) from 15 hospitals of Kaiser Permanente Northern California. We divided these six hours into 18 nonoverlapping 20-minute epochs. The power spectral density of each epoch was divided into three bands: low frequency (LF, 30–150 mHz), movement frequency (MF, 150–500 mHz), and high frequency (HF, 500–1000 mHz). We also estimated the LF/(MF+HF) ratio, the mean and standard deviation of the FHR signal, the approximate entropy (ApEn), and the deceleration capacity (DC). In our results, ApEn, the standard deviation, and DC showed a promising ability to detect risk of HIE as early as 120 minutes before birth, which gives enough leading time for timely interventions.

## 1. Introduction

The assessment of fetal wellbeing during labor is largely based on the visual assessment of fetal heart rate (FHR) and uterine pressure (UP) signals acquired with cardiotocography (CTG). Unfortunately, this is associated with large intra- and inter-subject variability. As an alternative, multiple studies have proposed analyzing other computerized FHR features to objectively quantify changes in the signal that could indicate fetal acidemia. Early detection of fetal compromise is particularly important for the prevention of hypoxic-ischemic encephalopathy (HIE), where timely cesarean section can avoid severe acidemia and subsequent HIE.

The availability of large databases of FHR signals has sparked the development of specialized machine learning classifiers that aim to improve the early detection of fetal compromise. However, it is expected that FHR is nonstationary over the course of labor. For this reason, we examined the temporal evolution of FHR features as labor progressed in normal fetuses and fetuses that were diagnosed with HIE. The knowledge of how these features vary throughout labor should allow classifiers to account for these changes and improve their performance.

## 2. Methods

This section describes the dataset, the methods for processing the FHR signals, and how the parameters of the distributions of fHRV features were estimated.

### 2.1. Fetal database

Our group has access to clinical and CTG signals collected in 15 Kaiser Permanente Northern California hospitals between 2011 and 2019. The study population consists of 246,968 singleton births of at least 35 weeks of gestational age. Of these, 357 had HIE, defined as blood pH < 7 or base deficit > 9 mmol/L, and clinical evidence of encephalopathy. We considered only subjects born vaginally to avoid the interruption of the natural progression of labor caused by cesarean delivery. We also limited our study group to subjects who had a cord blood gas or a neonatal blood gas within the first 120 minutes of life. This selection yielded two classes: 21,853 subjects with normal neonatal outcomes and normal blood gases and 163 subjects with HIE.

### 2.2. FHR pre-processing

The FHR signals in our database were recorded at bedside during the intrapartum period using CTG clinical monitors. In this study, we examined the last 6 hours of recordings before birth. We divided the FHR records into 18 nonoverlapping, 20-minute-long epochs. For simplicity, we will refer to epoch-N as the 20-minute epoch starting N minutes before birth. We only considered for analysis epochs with at least 80% valid samples.

The FHR signals were sampled at 4 Hz. We filled gaps shorter than 50 samples (12.5 s) using linear interpolation.

### 2.3. Fetal heart rate features

To characterize the temporal evolution of the distribution of the FHR signals, we estimated the mean (*FHR*_*μ*_) and the standard deviation of the FHR (*FHR*_*σ*_).

Power spectral density (PSD) features have been shown to have discriminatory information for fetal compromise [1]. For each epoch, we computed the PSD using the Lomb-Scargle periodogram, which can handle gaps in the signal [2]. Then, we estimated the power in the low frequency (LF, 30 – 150 mHz), movement frequency (MF, 150 – 500 mHz), and the high frequency (HF, 0.5 – 1 Hz) bands [1]. We also estimated the LF/(MF+HF) power ratio.

Measures of signal irregularity, such as the approximate entropy (ApEn) have been also shown to have discriminatory information of fetal compromise [1]. ApEn was estimated with an embedding dimension of 2 and a radius of 0.2 on the FHR decimated by a factor of 2. This choice of parameters provide good agreement between the decimated FHR signal and the beat-to-beat interval, which is not available with CTG [5]. ApEn was estimated using the ‘approximateEntropy’ function in Matlab.

Finally, we estimated the deceleration capacity (DC) of FHR. DC is a measure of the variation of the FHR signal downwards. Previous studies showed that DC has discriminatory information of fetal compromise [3]. We estimated DC using the toolbox provided in [3].

### 2.4. Temporal analysis of features

We estimated the features at each epoch-N across all the subjects for the normal and HIE classes. For each feature and epoch, we estimated its median and its 95% confidence interval (CI) though statistical resampling: we used 1,000 bootstrapping samples of the estimated features and estimated the median of each sample. Then we obtained the 2.5 and 97.5 percentiles to determine the span of the 95% CI of the median. We focused on the median because most of the distributions were skewed, and the median is more robust to skewness and outliers than the mean. Also, at each epoch, we used the Kolmogorov-Smirnov (KS) distribution test to test whether the normal and HIE features came from the same distribution.

## 3. Results

### 3.1. Number of subjects per epoch

**Figure 1.**
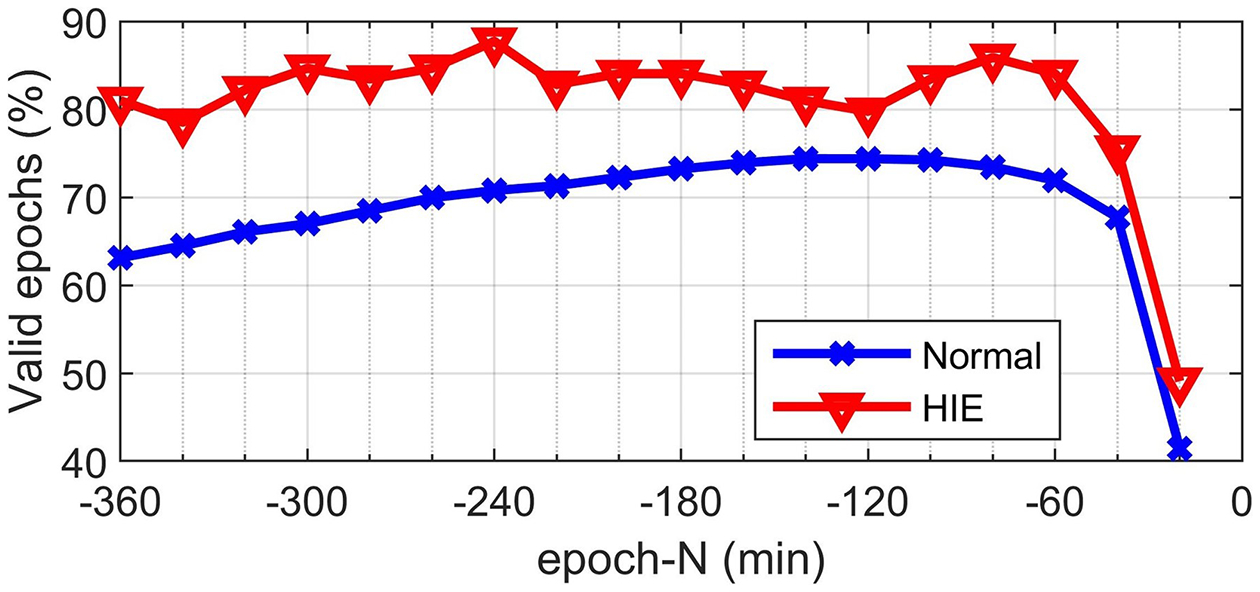
Proportion of normal (blue) and the HIE (red) subjects with valid epochs as a function of time.

Figure 1 shows the proportion of subjects with valid data for each epoch. A larger proportion of HIE subjects had valid epochs than normal subjects. There was a steep drop in the proportion of subjects with valid epochs at epoch-20. For this reason, the temporal analysis of features was restricted to epoch −360 to epoch-40.

### 3.2. Distribution of FHR features

**Figure 2.**
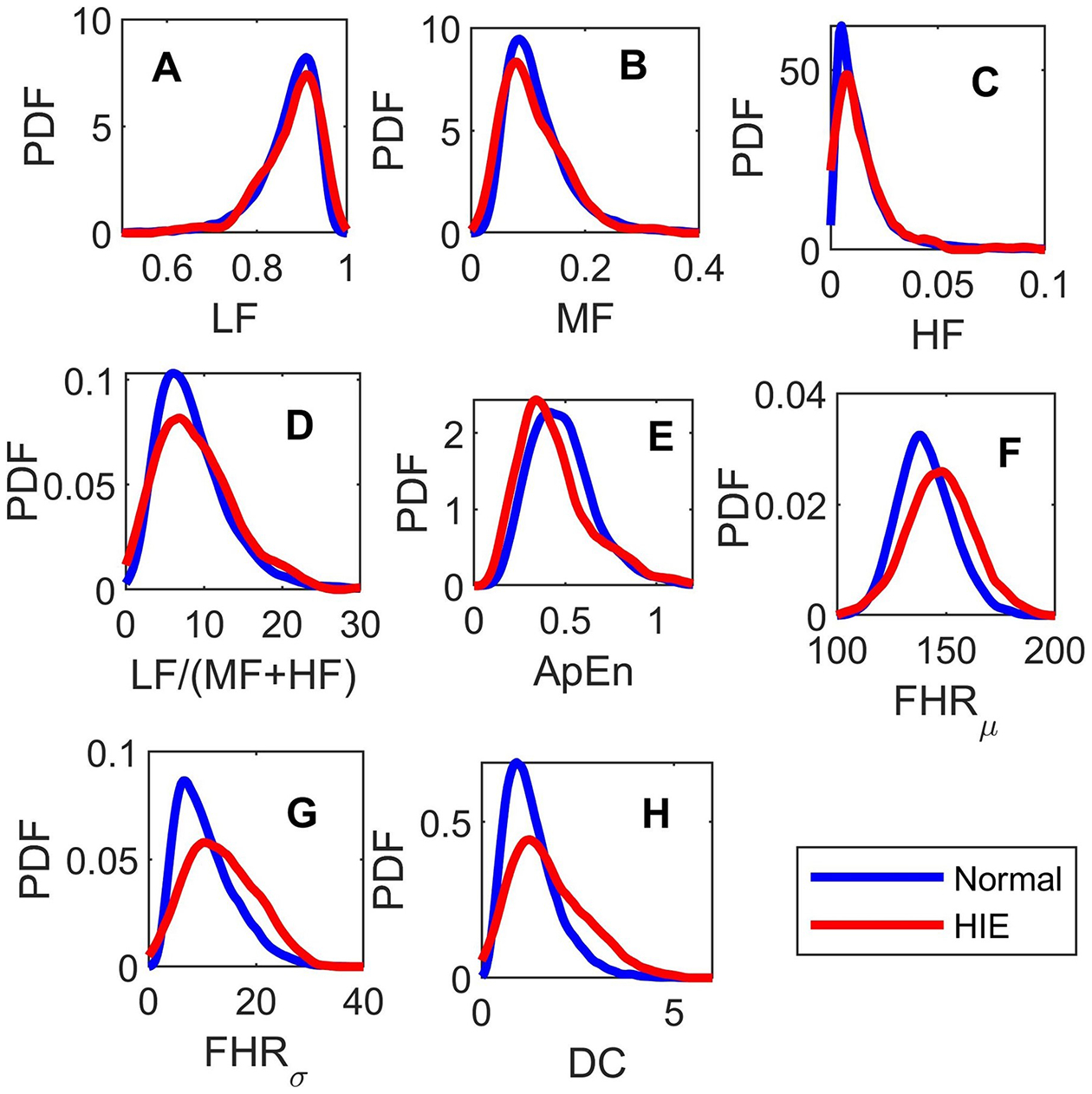
Kernel distributions of (A) LF, (B) MF, (C) HF, (D) LF/(MF+HF), (E) ApEn, (F)*FHR*_*μ*_, (G) *FHR*_*σ*_, and the (H) DC obtained for the normal (blue) and HIE (red) classes at epoch-100.

Figure 2 shows the nonparametric distributions of each feature estimated for epoch-100. For visualization, we used Gaussian Kernels to generate the continuous and smooth probability density functions. All distributions were skewed, which justifies the choice of analyzing their medians. In epoch-100, only four features showed significant difference between the normal (blue) and HIE (red) classes according to the KS test. These features were: ApEn, where the HIE distribution was shifted to the left of the normal distribution; *FHR*_*μ*_, *FHR*_*σ*_, and DC distributions were shifted to the right and broader than the those of the normal subjects.

### 3.3. Temporal evolution of FHR features

**Figure 3.**
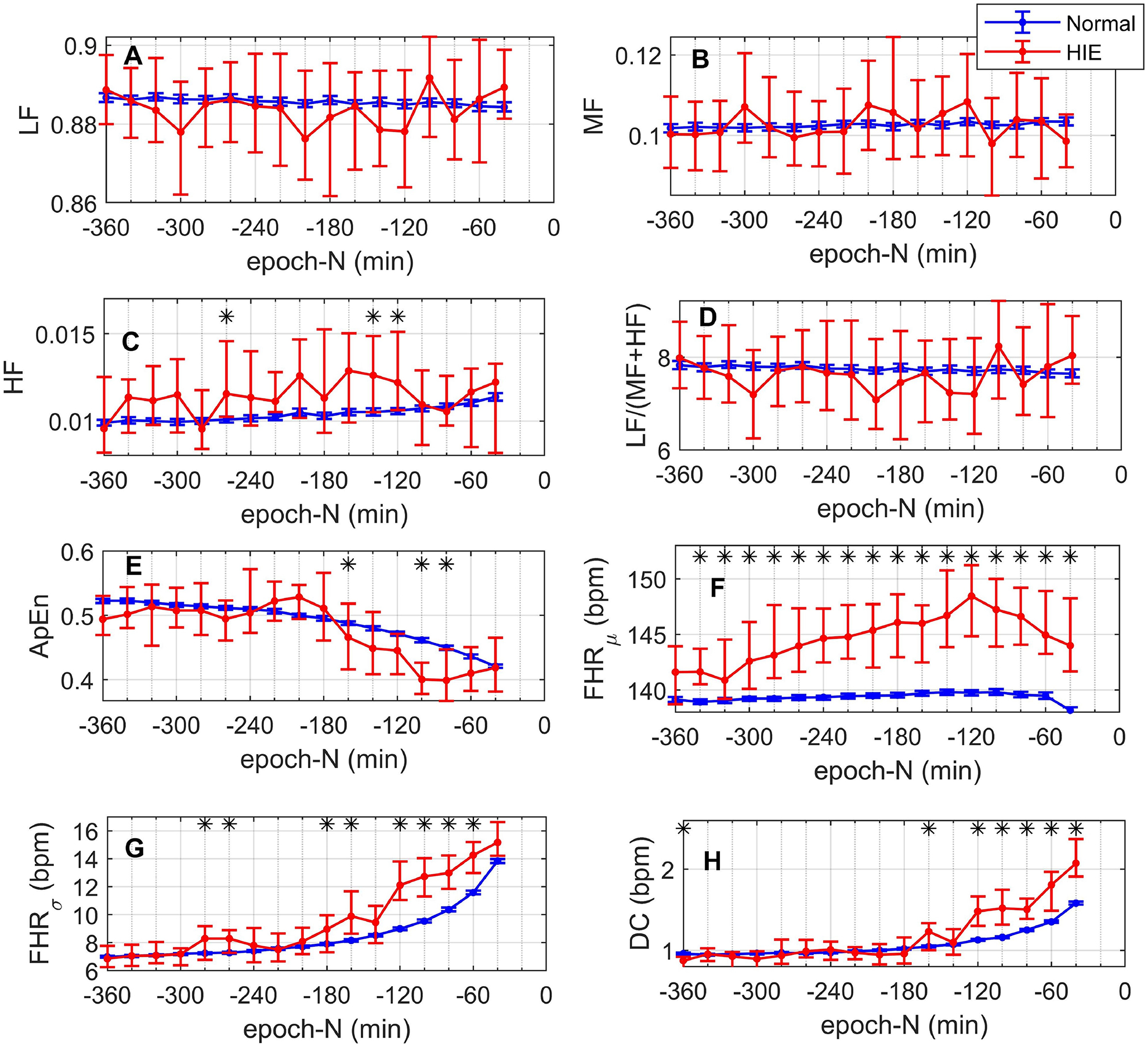
Temporal evolution of the median and its 95% CI of (A) LF, (B) MF, (C) HF, (D) LF/(MF+HF) ratio, (E) ApEn, (F) *FHR*_*μ*_, (G) *FHR*_*σ*_, and (H) DC for the normal (blue) and HIE (red) classes as functions of the epoch-N. The black asterisks indicate significant difference according to the Kolmogorov-Smirnov test.

Figure 3 shows the temporal evolution of the median, and its 95% CI, of the fHRV feature. For all features, the 95% CI of their median was larger for the HIE (red) than for the normal class (blue). The black asterisks indicate significant differences between normal and HI distributions according to the KS test. The results shown in Figure 3 can be summarized as follows:

#### PSD features:

Figure 3C shows a small increase in the HF of the normal class during the last 120 minutes before birth. HF is the only PSD feature that shows significant difference for normal and HIE distributions at epoch-260, −140, and −120. There was also a small decrease in the LF/(MF+HF) ratio of the normal class in Figure 3D. No trend can be recognized for the features of the HIE class or the LF and MF of the normal class.

#### ApEn:

Figure 3E shows that ApEn decreased as delivery approached for both normal and HIEs. These changes were most evident starting at epoch-200. ApEn shows significant differences for epoch-160, −100, and −80.

#### FHR_μ_:

Figure 3F shows that *FHR*_*μ*_ was quite stable for the normal class, decreasing at epoch-40. In contrast, for HIE, *FHR*_*μ*_ increased from epoch-360 to epoch-120 and then decreased. Moreover, *FHR*_*μ*_ was significantly larger for the HIE class from epoch-340 onward.

#### FHR_σ_:

Figure 3G shows that *FHR*_*σ*_ increased as delivery time approached for both normal and HIE classes. Furthermore, *FHR*_*σ*_ was larger for the HIE in epochs-280, −260, −180, −160, −120, −100, −80, and −60.

#### DC:

Figure 3H shows that DC increased in both normal and HIEs as delivery approached. Moreover, DC was significantly larger in the HIEs in epoch −160, and from epoch-120 onward.

## 4. Discussion

Our results showed that the median *FHR*_*σ*_ and DC rose as labor progressed. In addition, their distributions showed significant group differences in most of the epochs in the last 120 minutes before birth with higher levels in the HIE group. ApEn decreased with time and showed significantly lower levels in the HIE class in the epochs at 100 and 80 minutes before birth.

Hypoxic events, created by uterine contractions, or umbilical cord compression can lead to large and transient reductions of FHR known as decelerations [4] which in turn would cause high *FHR*_*σ*_ and DC. Acidemia of a level sufficient to cause HIE is thought to develop over time especially when clinical factors that cause hypoxemia intensify and exceed fetal compensatory mechanisms. Thus, the timing of the divergence of *FHR*_*σ*_ and DC measurements in these two groups is consistent with increasing frequency and strength of contractions, and cord compression seen at the end of labor. Nevertheless, the HIE group demonstrated significantly higher levels over many epochs. It is also clinically plausible that the lower ApEn levels, which are associated with neurological pathology, diverged later with lowest levels in the HIE group at epochs −100 and −80.

*FHR*_*μ*_ was consistently higher in the HIE group, beginning as early as epoch-340. This finding may represent association with other clinical risk factors such as the presence of maternal fever or chorioamnionitis that are known to increase susceptibility to hypoxemia. It may also represent a fetal compensatory mechanism needed to maintain oxygen delivery to the fetal central nervous system in an unfavorable environment and hence greater risk of later hypoxemia. These results are encouraging because they suggest that considering the evolution of these factors in combination may be useful in finding an optimal time for intervention to avoid HIE.

Finally, our results showed that the PSD features did not show much change over time or difference between the two classes. This does not agree with previous studies that showed that PSD features are discriminatory of fetal state and that they vary with time [1,5]. We believe different measurement techniques may explain this disparity. Signorini et al. analyzed the PSD of antepartum data, which does not present as many decelerations as the intrapartum signals analyzed in our study [1]. In the future, we will analyze the PSD of the FHR in baseline segments of the signal.

## 5. Conclusions

Our results showed that ApEn, *FHR*_*σ*_, and DC change the most during the progression of labor. Also, their distributions showed a promising ability to identify subjects at risk of developing HIE as early as 2 hours before labor. During intrapartum fetal monitoring, the time of delivery is unknown. Thus, distinguishing between the normal and HIE classes becomes a complicated problem. ApEn, *FHR*_*σ*_, and DC seem to be promising identifiers of fetal compromise, giving up to two hours of leading time to perform timely interventions such as emergency cesarean delivery. Finally, *FHR*_*μ*_ is likely to contain discriminatory information of HIE as early as epoch-340.
